# Dupuytren’s Disease of the Distal Interphalangeal Joint: A Systematic Review of Case Reports and Case Series

**DOI:** 10.3390/medicina62050903

**Published:** 2026-05-07

**Authors:** Ishith Seth, Sai-Vignesh Ashok, Omar Shadid, Raj Saini, Warren Rozen, Snehal Shah

**Affiliations:** 1 Department of Surgery, Peninsula Health, Melbourne 3199, Australia; ishithseth1@gmail.com (I.S.); raj_saini_2@sfu.ca (R.S.); warrenrozen@hotmail.com (W.R.); 2 Department of Surgery, Monash University, Melbourne 3168, Australia; snazzshah@hotmail.com; 3 Department of Plastics and Reconstructive Surgery, Peninsula Health, Melbourne 3199, Australia; vignesh.ashok.2605@gmail.com; 4 Department of Plastics and Reconstructive Surgery, Austin Hospital, Melbourne 3084, Australia

**Keywords:** digital deformity, distal interphalangeal joint, Dupuytren’s disease, limited fasciectomy, lateral digital cord

## Abstract

*Background and Objectives*: Dupuytren’s disease (DD) most commonly affects the palm and metacarpophalangeal/proximal interphalangeal joints; distal digital involvement at the distal interphalangeal joint (DIPJ) is uncommon and incompletely characterised. This systematic review summarises reported cases of DD involving the DIPJ, with or without proximal interphalangeal joint (PIPJ) involvement, focusing on clinical patterns, management, and reported outcomes. *Materials and Methods*: A systematic search of PubMed, Embase, and Scopus from database inception to July 2025 identified published case reports and case series describing DD confined to the DIPJ ± PIPJ of a single digit. Data were extracted on the demographics, digit involvement, anatomic features when reported, interventions, outcomes, complications, recurrence, and follow-up. *Results*: Nine studies reported 13 patients, published between 1991 and 2023. All patients were male (age range 25–79 years). The little finger predominated (10/13), followed by the ring (2/13) and index finger (1/13). When laterality was described, radial-sided distal cords were common. Surgical fasciectomy was performed in 11 cases and collagenase clostridium histolyticum (CCH) in 2 cases. Where postoperative correction was reported, outcomes were generally favourable; however, joint-specific range-of-motion outcomes and follow-up were inconsistently documented. Follow-up, when reported, ranged from 3 months to 3 years. One recurrence involving the PIPJ was reported 36 months after surgical management. No intraoperative neurovascular or tendon injuries were described, although adverse-event reporting was incomplete in some reports. *Conclusions*: Reported cases of DD involving the DIPJ most frequently involve the little finger in men. However, the available evidence is limited to a small number of selectively published case reports and series with incomplete outcome and follow-up reporting. These observations should therefore be interpreted cautiously, and comparative effectiveness or durability estimates cannot be established.

## 1. Introduction

Dupuytren’s disease (DD) is a benign fibroproliferative disorder affecting the palmar and digital fascia, defined by abnormal collagen deposition, myofibroblast proliferation, and the development of pathological cords [1,2]. These cords result in progressive flexion contractures of the fingers, most frequently involving the metacarpophalangeal (MCP) and proximal interphalangeal (PIP) joints [3,4]. The pretendinous and central cords are most implicated in these deformities, causing the characteristic clinical pattern observed in early-stage disease [5].

Contractures involving the distal interphalangeal (DIP) joint are uncommon. DIPJ involvement usually requires disease extension into the lateral digital or retrovascular cords, which are less often affected in DD and are located near neurovascular bundles. This anatomical complexity makes their involvement rare and surgically challenging. As a result, isolated DIPJ contractures are infrequently reported and are considered an atypical manifestation of the disease. The first documented cases of isolated DIPJ involvement were reported by Bellonias and Nancarrow in 1991, describing two middle-aged men with pathological cords affecting the DIPJ of the little finger following trauma [6]. The cords extended to the dorsum of the distal phalanx and nail bed, a pattern not previously recorded. Subsequent case reports have described similar presentations [7,8,9]. Rao et al. (2006) reported an isolated DIP contracture of the little finger [8], while Saleh et al. (2009) documented DD confined to the interphalangeal joints, with cord involvement on both the radial and ulnar aspects of the little finger [9]. In these cases, most patients were male and over 60 years of age, with predominant involvement of the little finger and the radial cord.

Because isolated DIPJ contracture in DD is rare and has not been comprehensively synthesised, a systematic review of the published literature is warranted. In patients with Dupuytren’s disease involving the distal interphalangeal joint of a single digit, with or without proximal interphalangeal joint involvement, what management strategies have been described in the literature and what clinical outcomes, including degree of contracture correction, complications, recurrence, and follow-up duration, have been reported? Improved understanding of these features may support earlier recognition and inform operative decision-making in patients with this atypical disease pattern.

## 2. Methods

### 2.1. Study Design

This systematic review was conducted in accordance with the Preferred Reporting Items for Systematic Reviews and Meta-Analyses (PRISMA) 2020 guidelines and summarised clinical patterns, management approaches, and reported outcomes, including degree of contracture, surgical and non-surgical interventions, complications, recurrence, and follow-up duration, using a narrative synthesis approach. Given the rarity of the condition under review, the included study designs were restricted to case reports and case series, as these represent the primary evidence base for isolated distal interphalangeal joint involvement in DD. The protocol was registered with the International Prospective Register of Systematic Reviews (PROSPERO) (ID: CRD420251274223).

### 2.2. Search Strategy

A systematic literature search was conducted across PubMed, Embase, and Scopus from database inception to July 2025. The search employed medical subject heading terms, Boolean operators, and free-text keywords to capture all relevant publications. Search terms included: “Dupuytren’s disease OR Dupuytren’s contracture” AND “distal interphalangeal joint OR DIPJ” AND “isolated OR atypical presentation.” No date restrictions were imposed in order to facilitate a complete synthesis of the available evidence. Additional studies were identified through manual screening of reference lists from included studies. The full search strategy is provided in Supplementary File S1 [10].

### 2.3. Study Selection

**Figure 1:**
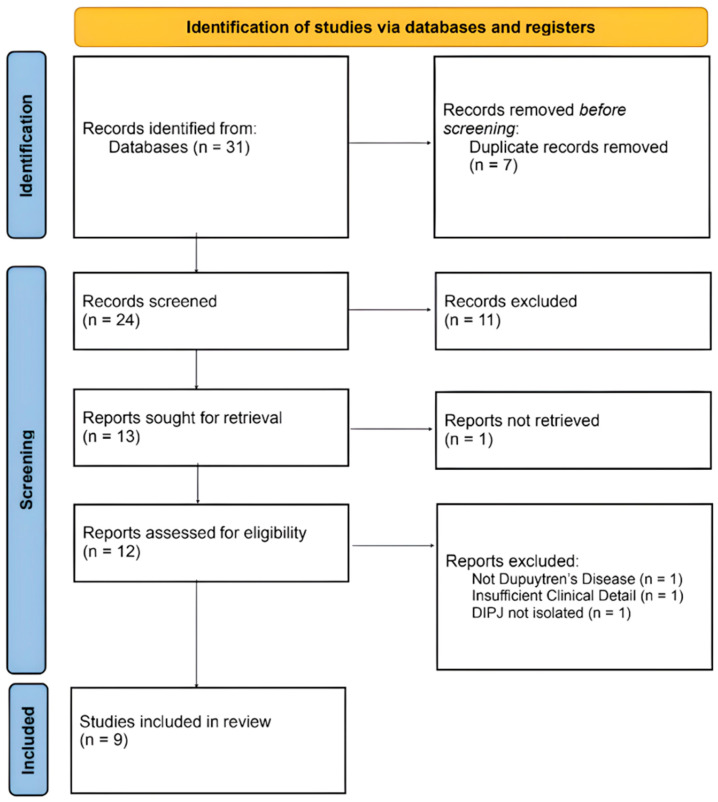
PRISMA guidelines.

Two independent reviewers (OS and SA) screened titles and abstracts using Covidence, a systematic review management software, applying the pre-specified inclusion and exclusion criteria. The full texts of potentially eligible studies were then retrieved and assessed for eligibility. Discrepancies between reviewers were resolved by discussion or by consulting a third reviewer (IS). The study selection process is summarised in a PRISMA flowchart (Figure 1).

Studies were eligible for inclusion if they reported cases of isolated DD involving the DIPJ, with or without associated PIP joint involvement. Cases were included if the original report described the contracture as DD based on clinical evaluation and/or intraoperative identification of a pathological fibrous cord consistent with Dupuytren pathology. Histopathological confirmation was not required because it was rarely reported in the case-based literature. Studies were excluded if they described DD extending to the MCP joints or cases limited to palmar cord involvement. Reviews, letters to the editor, expert opinions, and conference abstracts without full text were also excluded, as were non-English-language studies for which high-quality translations were unavailable.

### 2.4. Data Extraction

Data extraction was performed independently by two reviewers (OS and SA) using a predefined extraction template, with discrepancies resolved by consensus or consultation with IS. The extracted data included the study characteristics (author, year, country, and study design), patient demographics (age, sex, and relevant comorbidities), digit involved, degree of contracture, Tubiana stage where reported, management approach, intraoperative and postoperative complications, recurrence, clinical outcomes, and duration of follow-up. Extracted data were collated in a structured summary table (Table 1).

### 2.5. Quality Assessment

Risk of bias in included case reports and case series was independently assessed by two reviewers (OS and SA) using the Joanna Briggs Institute (JBI) Critical Appraisal Checklists for case reports and case series, respectively. Assessment focused on clarity of case definition, completeness of demographic and clinical information, adequacy of follow-up, and transparency of outcome reporting. Discrepancies in quality assessment were resolved by consensus among all three reviewers (OS, SA, and IS). Full details of the quality assessment are provided in Supplementary Table S1.

### 2.6. Data Synthesis

Given the small number of included cases and the inherent heterogeneity in outcome measurement and reporting across individual case reports and series, formal quantitative meta-analysis was not feasible. The data were therefore synthesised narratively, with findings reported descriptively across patient demographics, anatomical cord distribution, management strategies, and clinical outcomes.

## 3. Results

### 3.1. Characteristics of the Included Studies

Nine studies reporting thirteen patients (1991–2023) with DD involving the DIPJ of a single digit were identified (Table 1, Figure 1). To improve interpretability, cases were considered in two subgroups: isolated DIPJ involvement and combined PIPJ/DIPJ involvement. These rare cases affected only males and mostly involved the little finger. Where cord laterality was described, radial-sided involvement was common. Of the thirteen cases, ten were in the little finger, two in the ring finger, and one in the index finger.

Tubiana stages in these cases ranged from I to III. In isolated DIPJ cases, deformity ranged from 30° to 80° and was generally stage I or II. Stanley and Cavallo (2020) reported Stage I deformities in the right little finger (45°) and index finger (30°) [13]. Stage II, the largest subgroup, included DIPJ contractures of 50–70° [14] and one 60° fixed DIPJ contracture [6]. Stage III deformities appeared only in cases with both PIP and DIPJ involvement, such as Karasoy et al. (2012) (75° DIPJ, 30° PIPJ) [11], Saleh et al. (2009) (70° DIPJ, 50° PIPJ) [9], and Tonkin and Bellity (2016) (60° DIPJ, 50° PIPJ) [15], in whom total contracture reached 120°. These findings suggest that the highest Tubiana stages in this group reflect dual-joint rather than isolated DIPJ deformity.

All reported patients were male, aged 25 to 79 years. The two youngest patients were 25 [15] and 28 years old [14], while the oldest were 79 (Saleh et al. [9]) and 75 years old (Rao et al. [8]). Presentation at 25 years of age is clinically notable due to the epidemiological atypicality of DD at this age. However, the available evidence is insufficient to determine whether early onset predicts recurrence or progression within this confined-joint phenotype. The Stanley and Cavallo (2020) series represents the largest cohort to date and describes four patients with isolated DIPJ involvement in three little fingers and one index finger, with contractures ranging from 30° to 70° (Tubiana stages I–II) [13].

### 3.2. Management and Outcomes

Surgical management was utilised in 11 of 13 cases [6,8,9,11,13,14,15,16], with fasciectomy consistently resulting in full or near-full extension. Where specified, incision approaches included both lateral and longitudinal incisions (Stanley and Cavallo [13]). No intraoperative neurovascular or tendon injuries were described; however, adverse-event reporting was incomplete in some reports. However, this finding should be interpreted with caution because of reliance on case-level reporting. Two cases were managed with CCH injection: Mehdi et al. (2019) [12] and one patient in Stanley and Cavallo (2020) [13], both achieving short-term correction; direct comparison with surgical outcomes is not possible from the available case-level evidence. The long-term durability of CCH in this anatomical zone was not assessed.

Only one recurrence was documented among patients undergoing surgical fasciectomy: Bellonias et al. [6] reported a 30° PIPJ recurrence at three years in a combined PIPJ/DIPJ case, with no recurrence of DIPJ involvement. Where specified, follow-up ranged from three months to three years, with the longest documented follow-up reported by Bellonias et al. [6] and Tonkin and Bellity [15]. Follow-up duration was not reported in Rao et al. [8], Yamada et al. [16], and for several patients in Stanley and Cavallo [13], including one CCH-treated patient. This lack of consistent follow-up limits confidence in recurrence estimates across a substantial proportion of the cohort.

### 3.3. Quality Assessment

Overall, reporting quality was high across the included studies (Table A1). Most studies met the pre-specified reporting domains, with seven scoring 8 out of 8 and two scoring 7. However, Rao et al. (2006) [8] and Tonkin and Bellity (2016) [15] did not report adverse or unanticipated events. Demographics, clinical history, presentation, intervention details, and post-intervention status were consistently reported.

The main limitation was the lack of documented diagnostic and assessment methods. While PRISMA 2020 Checklist scores (Supplementary File S1) indicate thorough reporting, the certainty of evidence remains limited by case-level study designs, publication bias, inconsistent follow-up, and the inability to estimate comparative effectiveness or recurrence rates precisely.

## 4. Discussion

This systematic review identified nine studies reporting 13 cases of DD involving the DIPJ. DIPJ-dominant DD showed a consistent pattern of male predominance, frequent little-finger involvement, and radial-sided distal cord disease. Isolated DIPJ cases were generally Tubiana stage I–II, whereas stage III deformity occurred only in combined PIPJ/DIPJ cases, suggesting that higher severity reflected additive dual-joint contracture rather than a distinct aggressive subtype. Reported outcomes were generally favourable after treatment, although postoperative ROM, complications, and follow-up were inconsistently documented.

The clinical relevance of these findings is primarily diagnostic and operative. Clinically, DIPJ flexion contracture attributable to a discrete distal cord can represent DD even when classic palmar findings are absent (general background); however, palmar involvement was not systematically reported in the included cases. Failure to recognise this presentation risks diagnostic delay, inadequate counselling, and increased operative risk if distal digital anatomy is managed as a straightforward extension of the palmar technique [17,18].

### 4.1. Anatomic Basis and Phenotype Interpretation

DIPJ involvement occurs when disease in the lateral digital and retrovascular compartments produces flexion contracture at the distal joint. These fascial structures are close to the terminal neurovascular bundle, making distal cord excision surgically complex [6,7]. The frequent observation of radial-sided cords suggests that DIPJ-dominant DD typically follows established distal fascial pathways, rather than representing random or ectopic fibrosis [5,19].

Two patients presented at an unusually young age: 25 years and 28 years [14,15]. Early onset is recognised in DD diathesis and is associated with higher recurrence or progression rates [20,21]. The biologic progression and significance of early DIPJ involvement is unclear due to limited data.

### 4.2. Operative Considerations

Limited fasciectomy is the best-supported intervention in the literature studied. All 11 surgically treated patients achieved full or near-full correction after excision of a discrete distal cord [6,8,9,11,13,14,15,16]. No intraoperative complications, including neurovascular injury, were reported. However, the absence of reported complications in this small case-based literature review should not be interpreted as an absence of risk. In the broader DD literature, reflex sympathetic dystrophy/complex regional pain syndrome (CRPS) is a recognised and clinically important postoperative complication after treatment, underscoring the need for counselling, early recognition, and appropriate postoperative monitoring [5,17]. This finding needs cautious interpretation because of reliance on case-level reporting and the lack of documented diagnostic and assessment methods in the studies.

These findings suggest that distal cords may course near or displace the terminal neurovascular bundle, especially with spiral components [19,22]. The confined soft-tissue envelope demands careful dissection, precise neurovascular identification, and readiness to extend exposure if planes are unclear [22]. Counsel patients accordingly: the technical demands for distal cord excision often exceed those for comparably sized proximal dissections.

When evaluating an isolated fixed DIPJ contracture, expand your differential beyond typical palmar DD: consider tendon imbalance, arthritis, or dermal scarring. If the diagnosis is uncertain, use high-resolution ultrasound to localise the cord and delineate its relationship to the neurovascular bundle before surgery [18,23].

### 4.3. Collagenase in DIPJ-Dominant Disease

CCH has well-established randomised trial evidence for MCP and PIP contractures in DD [17,22]. In DIPJ-dominant disease, the evidence is limited to two cases in this review [12,13]. Both achieved short-term correction without reported complications, but follow-up was insufficient to assess durability, which is a significant gap in a condition where recurrence risk accumulates over time.

Because the distal digit has a confined soft-tissue envelope and closely apposed terminal neurovascular structures, CCH injection in this region should not be managed as a simple extension of palmar protocols [18,19,23,24,25,26]. Appropriate case selection (a clearly palpable cord), meticulous injection technique, and structured post-procedure therapy are essential; however, existing reports remain too limited to draw broader safety inferences [26].

### 4.4. Limitations

Several limitations constrain the certainty of this review. Most importantly, diagnostic heterogeneity cannot be excluded. Isolated DIPJ contracture has a broader differential than typical palmar DD. As a result, misclassification of non-DD causes could occur and inflate apparent treatment success. This limitation should be considered when using these findings in clinical practice.

Beyond diagnostic uncertainty, the evidence base is restricted to case reports and small case series, precluding pooled estimates of recurrence, complication rates, or comparative effectiveness [27]. Follow-up was often absent or brief, completely unreported in three studies and limited to 3 months in two others, limiting all durability claims. Additionally, ROM outcome reporting was absent in three cases [8,9,16].

An important limitation of the available literature is the short and inconsistently reported duration of follow-up. Follow-up data were available for only 7 of the 13 reported cases, with the longest reported duration being 3 years. This timeframe is substantially shorter than the intervals over which recurrence is commonly evaluated in the broader Dupuytren literature, where standardised joint-specific recurrence reporting has been recommended and meaningful recurrence continues to be observed on 5-year-and-longer follow-up [28,29,30,31]. Consequently, the single recurrence reported in the included literature should not be interpreted as reflecting a low recurrence rate. Rather, the available data are insufficient to meaningfully assess long-term durability or recurrence following treatment of DIP joint involvement in Dupuytren’s disease [28,29,30,31]. Longer-term follow-up in future reports will be necessary to better characterise recurrence patterns in this atypical disease presentation.

Publication bias is likely: atypical presentations with favourable outcomes are preferentially reported, while failures and neurovascular complications may be underrepresented [32]. The cohort is small (*n* = 13), and although high JBI checklist scores indicate generally complete reporting across most domains, the absence of documentation of diagnostic methodology was a universal finding. The overall certainty of evidence is low, and conclusions should be interpreted as hypothesis-generating rather than definitive estimates of effectiveness, durability, or complication risk [27]. Lastly, formal pooling of results was not possible given the heterogeneity and small size of the included studies, precluding quantitative estimates of recurrence, complication rates, or comparative effectiveness.

### 4.5. Future Directions

Progress in understanding DIPJ-dominant DD requires standardised reporting and multi-institutional data capture. At minimum, future case reports should document explicit diagnostic criteria and the differential diagnoses considered, joint-specific extension deficits recorded separately for the MCP, PIP, and DIPJ, cord type and anatomical compartment with neurovascular relationship, standardised functional and patient-reported outcomes, and recurrence definitions with time-stamped longitudinal follow-up [28].

Imaging–anatomic correlation is an actionable research avenue: high-resolution ultrasound and MRI can help localise isolated digital cords and anticipate neurovascular proximity, potentially improving both diagnostic confidence and operative planning in atypical presentations [18]. Prospective registries, rather than randomised trials which are unlikely to be feasible for this rare phenotype, could enable pragmatic comparisons of limited fasciectomy, percutaneous needle aponeurotomy, and CCH in matched presentations, while capturing adverse events systematically [32].

## 5. Conclusions

DIPJ-dominant DD represents a rare but reproducible clinical phenotype characterised in the published literature by male predominance, frequent little-finger involvement, and distal cords, often described along the radial aspect, producing clinically meaningful flexion contracture at the terminal joint. Across the limited available evidence (13 patients), fasciectomy most consistently achieved full or near-full correction, while CCH has only case-level support with short-term improvement and uncertain durability. Recognition of this distal presentation is essential for accurate diagnosis, appropriate counselling regarding recurrence and neurovascular risk, and operative planning within the confined distal digit. Given the low certainty of evidence, future progress depends on standardised reporting with joint-specific outcomes, explicit diagnostic criteria, neurovascular-anatomic documentation, and longitudinal follow-up through multi-institutional registries to better define durability, complications, and comparative effectiveness.

## Figures and Tables

**Table 1:** Summary of all included case reports.

Author (Year)	Patient Demographics	Digit(s) Involved	Degree of Contracture	Management	Reported Basis for Diagnosis	Outcomes	Follow-Up Duration
Bellonias et al. 1991 [6]	49-year-old male	Right little finger (isolated to radial DIPJ)	Stage 2 Tubiana (60° fixed contracture)	Surgical fasciectomy	Clinical and Intraoperative findings	Full ROM. Nil complications or recurrence	3 years
Bellonias et al. 1991 [6]	67-year-old male	Right little finger (confined to radial DIP and PIPJ)	Stage 2 Tubiana (40° fixed PIPJ, 40° fixed DIPJ)	Surgical fasciectomy	Clinical and Intraoperative findings	Nil complications. Nil recurrence of DIPJ, however after 3 years 30° PIPJ recurrence evolved	3 years
Karasoy et al. 2012 [11]	51-year-old male	Right little finger (confined to radial DIPJ and PIPJ)	Stage 3 Tubiana (75° at DIPJ, 30° at PIPJ)	Surgical fasciectomy	Clinical and Intraoperative findings	Full correction. Nil recurrence or complications	3 months
Mehdi et al. 2019 [12]	61-year-old male	Right little finger (isolated to radial DIPJ)	Stage 2 Tubiana (55–80°)	Collagenase injection	Clinical	Significant correction (10–80° active range, 10° at rest—can be corrected to 0° with assistance). Nil complications or recurrence	5 months
Rao et al. 2006 [8]	75-year-old male	Right little finger (isolated to radial DIPJ)	—	Surgical fasciectomy	Clinical and Intraoperative findings	Nil recurrence	—
Saleh et al. 2009 [9]	79-year-old male	Right little finger (confined to PIP and DIPJ, both radial and ulnar aspects)	Stage 3 Tubiana (fixed flexion PIPJ 50°, DIPJ 70°)	Surgical fasciectomy	Clinical and Intraoperative findings	“Good clinical outcome”	3 months
Stanley & Cavallo 2020 [13]	74-year-old male	Right little finger (isolated to DIPJ)	Stage 1 Tubiana (45° flexion deformity)	Surgical fasciectomy via lateral approach	Clinical and Intraoperative findings	Full extension achieved post-excision. Nil complications or recurrence reported	Not specified
Stanley & Cavallo 2020 [13]	56-year-old male	Left little finger (isolated to DIPJ)	Stage 2 Tubiana (70° flexion deformity)	Surgical fasciectomy via longitudinal incision	Clinical and Intraoperative findings	Full extension achieved after excision and manipulation. Nil complications or recurrence reported	Not specified
Stanley & Cavallo 2020 [13]	67-year-old male	Left little finger (isolated to DIPJ)	Stage 2 Tubiana (50° flexion deformity)	Collagenase Injection	Clinical	Successful cord rupture with full extension. Nil complications or recurrence reported	Not specified
Stanley & Cavallo 2020 [13]	54-year-old male	Right index finger (isolated to DIPJ)	Stage 1 Tubiana (30° flexion deformity)	Surgical fasciectomy	Clinical and Intraoperative findings	Full extension achieved. Nil complications or recurrence reported	Not specified
Takase et al. 2010 [14]	28-year-old male	Right ring finger (isolated to radial DIPJ)	Stage 2 Tubiana (50–70°)	Surgical fasciectomy	Clinical and Intraoperative findings	Full ROM (0–80° active range). Nil complications or recurrence	1 year
Tonkin & Bellity 2016 [15]	25-year-old male	Left little finger (confined to radial PIPJ and DIPJ)	Stage 3 Tubiana (60° at DIPJ, 50° at PIPJ)	Surgical fasciectomy	Clinical and Intraoperative findings	Full correction. Nil recurrence or complications	3 years
Yamada et al. 2023 [16]	34-year-old male	Right ring finger (isolated to DIPJ)	—	Surgical fasciectomy	Clinical and Intraoperative findings	Nil complications or recurrence	—

## Data Availability

No new data were created or analyzed in this study.

## Supplementary Materials

The following supporting information can be downloaded at https://www.mdpi.com/article/10.3390/medicina62050903/s1, File S1: PRISMA 2020 Checklist for systematic reviews.

## Abbreviations

The following abbreviations are used in this manuscript:

DD	Dupuytren’s disease
DIPJ	Distal interphalangeal joint
DIP	Distal interphalangeal
PIPJ	Proximal interphalangeal joint
PIP	Proximal interphalangeal
MCP	Metacarpophalangeal
PRISMA	Preferred Reporting Items for Systematic Reviews and Meta-Analyses
JBI	Joanna Briggs Institute
CCH	Collagenase clostridium histolyticum
ROM	Range of motion
MRI	Magnetic resonance imaging

## Appendix A

**Table A1:** Quality Assessment.

Study (Year)	Demographics Clearly Described	History as Timeline	Clinical Condition on Presentation	Diagnostics/Assessment Methods Described	Intervention Clearly Described	Post-Intervention Condition Described	Adverse/Unanticipated Events Reported	Takeaway Lessons Provided	Overall Score
Bellonias & Nancarrow (1991) [6]	Yes	Yes	Yes	No	Yes	Yes	Yes	Yes	8
Karasoy (2012) [11]	Yes	Yes	Yes	No	Yes	Yes	Yes	Yes	8
Mehdi et al. (2019) [12]	Yes	Yes	Yes	No	Yes	Yes	Yes	Yes	8
Rao et al. (2006) [8]	Yes	Yes	Yes	No	Yes	Yes	No	Yes	7
Saleh et al. (2009) [9]	Yes	Yes	Yes	No	Yes	Yes	Yes	Yes	8
Stanley and Cavallo (2020) [13]	Yes	Yes	Yes	No	Yes	Yes	Yes	Yes	8
Takase et al. (2010) [14]	Yes	Yes	Yes	No	Yes	Yes	Yes	Yes	8
Tonkin & Bellity (2016) [15]	Yes	Yes	Yes	No	Yes	Yes	No	Yes	7
Yamada et al. (2023) [16]	Yes	Yes	Yes	No	Yes	Yes	Yes	Yes	8
