# Supplementary figures and images for: Dupuytren’s Disease of the Distal Interphalangeal Joint: A Systematic Review of Case Reports and Case Series
Source: Medicina (Kaunas). 2026 May 7;62(5):903. doi: 10.3390/medicina62050903 (PMC13208662; doi:10.3390/medicina62050903)

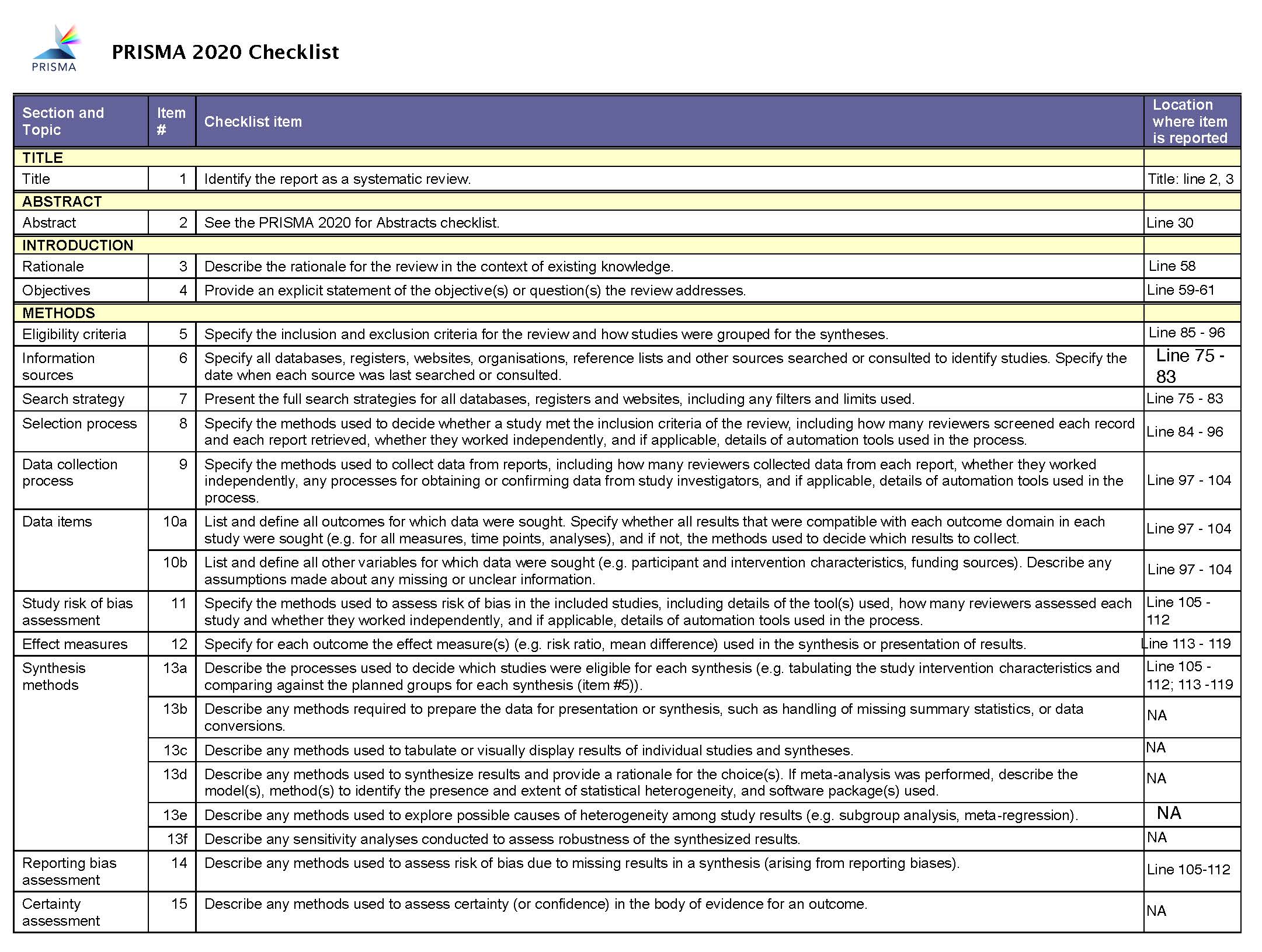

Supplement: Supplementary file 1 [file medicina-62-00903-s001.zip › medicina-4211865-supplementary-1.jpg]

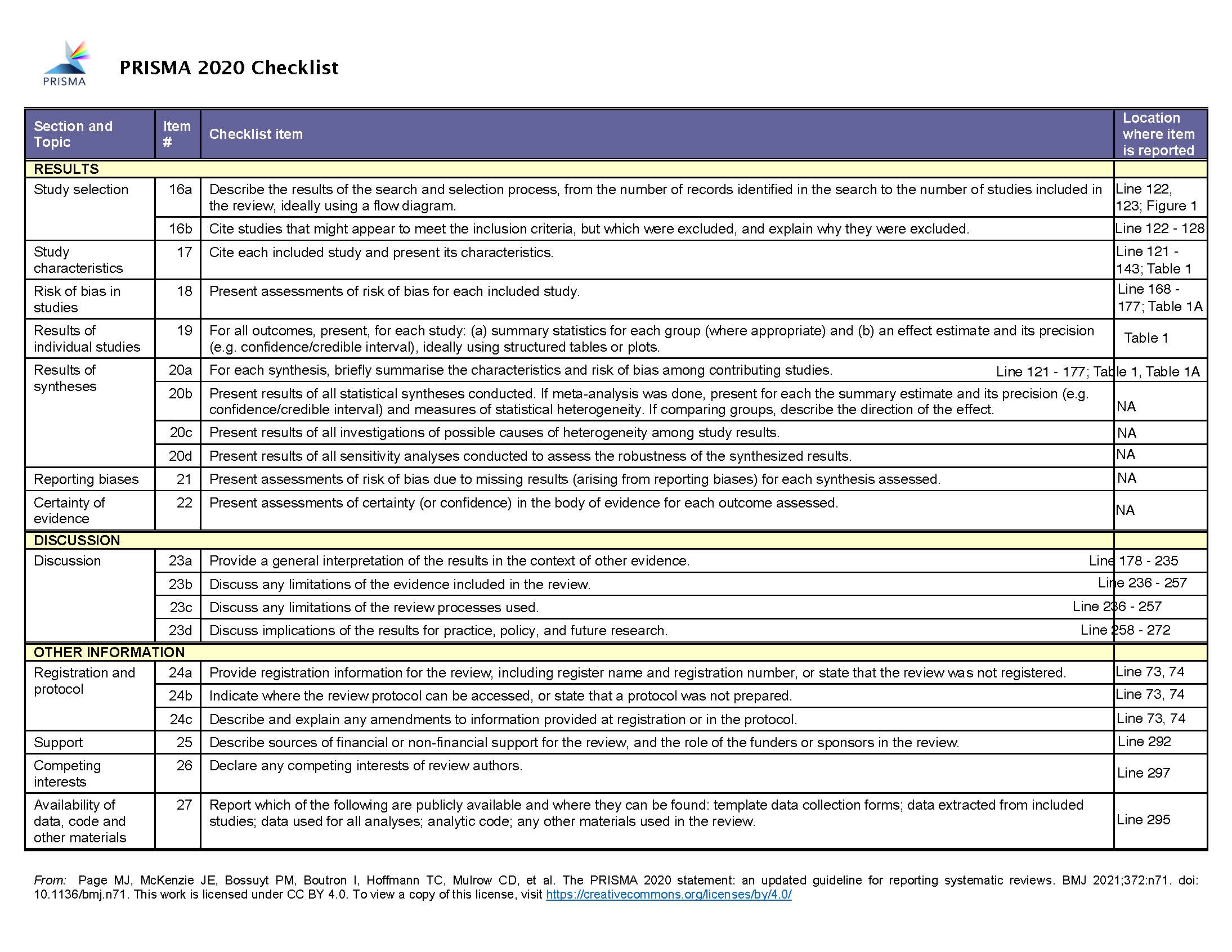

Supplement: Supplementary file 1 [file medicina-62-00903-s001.zip › medicina-4211865-supplementary-2.jpg]
